# Evaluating racial disparities in central-line–associated bloodstream infections for Tennessee hospitals, 2018–2021

**DOI:** 10.1017/ash.2023.286

**Published:** 2023-09-29

**Authors:** Simone Godwin, Erika Kirtz, Christopher Wilson

## Abstract

**Background:** Central-line–associated bloodstream infections (CLABSIs) significantly burden the US population and healthcare system. Reporting facilities in Tennessee consistently omit race and ethnicity data in the NHSN despite having the option to enter. Racial and ethnic disparities are well documented across many health outcomes, including patient safety. CLABSIs were compared among 3 racial groups to better understand the impact of race on CLABSI incidence in Tennessee. **Methods:** CLABSI data from NHSN were linked with records from the TN Hospital Discharge Data System (HDDS) for 2018–2021. A multivariable linear regression model was used to determine relative risk (RR) between racial groups for contracting a CLABSI after controlling for confounding variables including Charlson comorbidity index (CCI) and social vulnerability index (SVI) scores. Statistical significance was set at *P* < .05. Data linkage and statistical analyses were performed in SAS version 9.4 software. **Results:** In Tennessee between 2018 and 2021, 342 (17.2%) of the 1,980 CLABSI events had race documented, and no ethnicity variables exist in the NHSN. The data linkage process yielded a 72% match (1,426 CLABSIs). The remaining 28% were excluded from the analysis. Per 1,000 central-line days (CL days) for all races, white patients had the highest CLABSI rate (17.5), followed by Black patients (1.36), and Native American or Alaskan Native patients (0.68). Per 1,000 admissions by race, Black patients had a higher CLABSI rate (1.26) than Native American/Alaskan Native patients (0.85) and white patients (0.75). The risk of contracting a CLABSI was 79% higher in Black patients than in white patients (RR, 1.79; 95% CI, 1.55–2.07; *P* < .0001) when controlling for CCI, age group, and SVI. **Conclusions:** These results suggest that racial disparities between Black and white patients are present in Tennessee hospitals regarding CLABSIs. Although most CLABSI events were linked to HDDS patients, there were limitations in the ability to match all cases and calculate CL days by race. This study highlights the need for complete race and ethnicity data in the NHSN. Further studies should examine infection types at the regional and facility levels to target interventions for reducing HAI inequities in Tennessee.

**Disclosures:** None

